# RURAL-URBAN DIFFERENCES IN DIGITAL HEALTH USE OF MEDICARE BENEFICIARIES

**DOI:** 10.1093/geroni/igad104.3264

**Published:** 2023-12-21

**Authors:** Shannon Power, Hyesu Yeo

**Affiliations:** University of Georgia, Athens, Georgia, United States; University of Georgia, Athens, Georgia, United States

## Abstract

Growing reliance on digital healthcare appears to marginalize rural older adults with limited technology access and experience. Existing research explores telehealth uptake of Medicare beneficiaries but does not consider the role of locality in digital health use (DHU). Using the 2021 National Health and Aging Trends Study data, this study compares rural-urban Medicare beneficiaries’ DHU (weighted N=18,729,829). Informed by the Senior Technology Acceptance Model, multiple linear regressions were performed on rural and urban models separately, including DHU, social determinant, and technology readiness variables. Participants in this study were predominantly White, female, and in their 70s. In the final model, men were more likely to use DHU than women in both rural-urban samples (β=.13, β=.08, p<.05, respectively). Tablet access was the only significant device for DHU among rural residents (β=.18, p<.05), while computer access (β=.12, p<.05) was the most significant among urban residents. Online grocery shopping was the most predictive prior technology experience of DHU for rural dwellers (β=.24, p<.05), while online banking led for urban dwellers (β=.21, p<.05). These unique differences between rural-urban samples can inform research on how to specifically design and implement digital interventions to increase rural older adult uptake. Healthcare workers can emphasize similarities between DHU and older adults’ prior technology experience to build perceived ease of use. Expanding opportunities for rural older adults to interact with technology in daily life builds experience which best predicts DHU. Policymakers should consider the impact of locality and involve aging community stakeholders to advance equitable DHU in the digital age.

